# Ischemic territorial mismatch due to posterior cerebral artery occlusion illustrated by the FLAIR ivy sign in moyamoya disease

**DOI:** 10.1007/s11604-026-02012-7

**Published:** 2026-05-20

**Authors:** Shunji Mugikura, Naoko Mori

**Affiliations:** 1 Department of Diagnostic Radiology, Graduate School of Medicine, Tohoku University, Seiryo-machi, Aoba-ku, Sendai, 980-8574 Japan; 2 Department of Radiology, Graduate School of Medicine, Akita University, 1- 1-1 Hondo, Akita, 010-8543, Japan

**Keywords:** Moyamoya disease, Posterior cerebral artery, Collateral circulation, Magnetic resonance imaging, FLAIR ivy sign, Cerebral ischemia

## Abstract

Cerebral infarctions in moyamoya disease frequently do not conform to anatomical arterial territories. This phenomenon is closely associated with delayed steno-occlusive involvement of the posterior cerebral artery (PCA) following progression of internal carotid artery disease, which compromises PCA-derived leptomeningeal collateral circulation that had previously supported the anterior circulation. Although PCA involvement is common in moyamoya disease, the hierarchical vulnerability of PCA-derived collateral pathways and their relationship to the fluid-attenuated inversion recovery (FLAIR) ivy sign remain incompletely understood. This pictorial essay illustrates a hypothesis-generating conceptual framework of ischemic territorial mismatch caused by PCA steno-occlusion and clarifies the hemodynamic significance and clinical utility of the FLAIR ivy sign. PCA-derived leptomeningeal collateral circulation consists of two functionally distinct pathways: superficial collaterals supplying the middle cerebral artery (MCA) territory and deep collaterals supplying the anterior cerebral artery (ACA) territory via the posterior pericallosal artery. Because superficial collaterals traverse long cortical distances, they are more vulnerable to progressive PCA steno-occlusion than deep collateral pathways. Functional insufficiency of superficial collaterals may result in selective hemodynamic compromise of the MCA territory, while the PCA and ACA territories remain relatively preserved, producing ischemic territorial mismatch. Representative imaging findings from a pediatric cross-sectional case and an adult longitudinal case are presented to demonstrate this hemodynamic framework. Across the presented cases, the FLAIR ivy sign tends to preferentially appear in the MCA territory during superficial collateral insufficiency. In the longitudinal case, progression of PCA steno-occlusion was accompanied by emergence of the ivy sign in the MCA territory, which resolved after successful revascularization, highlighting its dynamic and reversible nature as a marker of hemodynamic compromise. In conclusion, ischemic territorial mismatch reflects hierarchical vulnerability of PCA-derived leptomeningeal collaterals. The FLAIR ivy sign provides a practical, noninvasive imaging marker of superficial collateral decompensation and hemodynamic stabilization after revascularization, offering guidance for longitudinal follow-up.

## Introduction

Cerebral infarctions in moyamoya disease often exhibit atypical distributions that do not conform to anatomical arterial territories. Such patterns are closely associated with delayed steno-occlusive changes of the posterior cerebral artery (PCA) that occur following progression of internal carotid artery (ICA) disease, leading to progressive insufficiency of PCA-derived collateral circulation that had previously compensated for the anterior circulation [1–3]. Notably, occlusive involvement of the PCA develops in approximately 45% of affected hemispheres [1, 2].

**Fig. 1:**
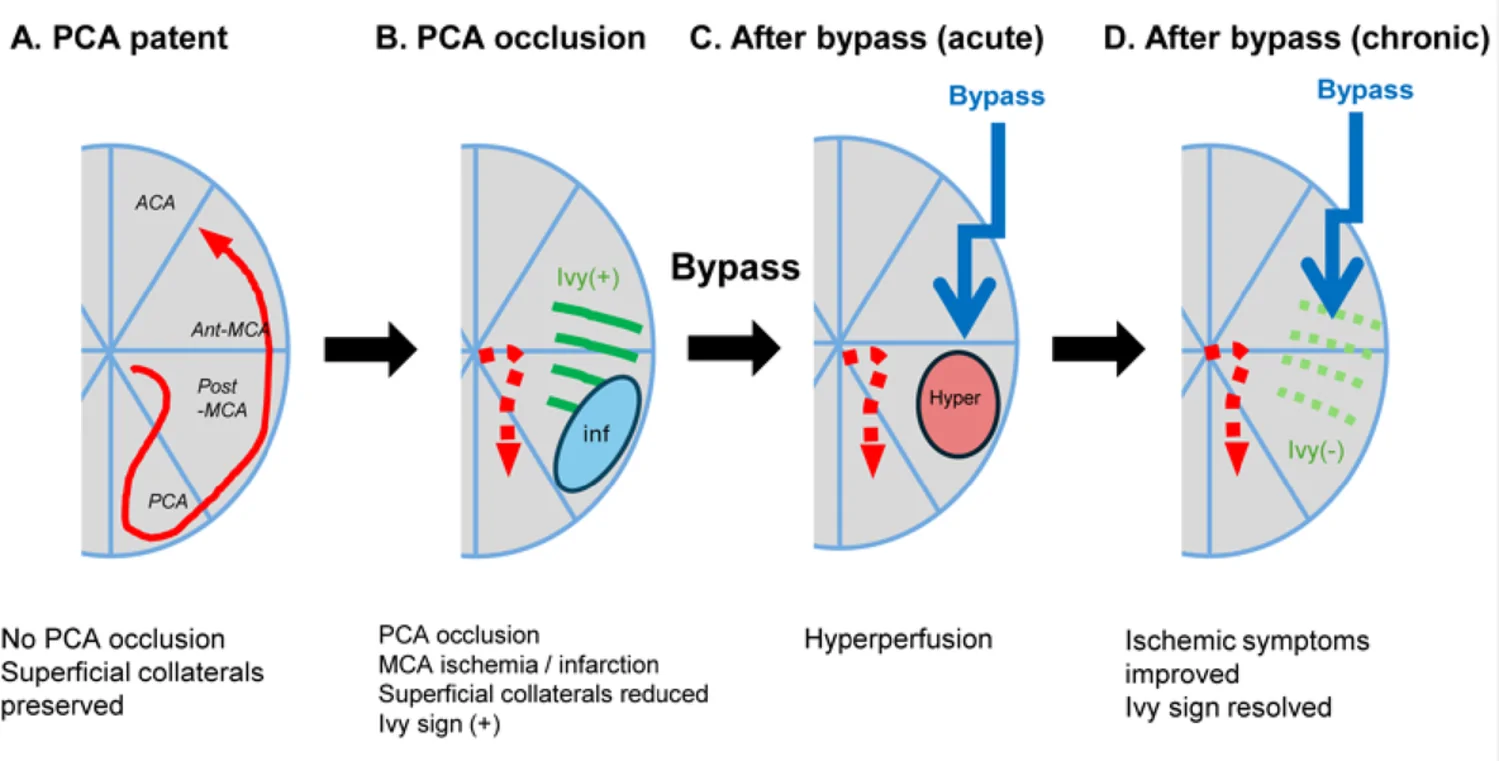
Conceptual schematic illustrating ischemic territorial mismatch associated with posterior cerebral artery involvement in moyamoya disease. Figure 1 illustrates a generalized hemodynamic framework underlying ischemic territorial mismatch related to posterior cerebral artery (PCA) involvement in moyamoya disease. **A** Compensated phase without PCA occlusion: When the PCA remains patent, PCA-derived superficial leptomeningeal collateral flow adequately supports perfusion of the anterior circulation, including the middle cerebral artery (MCA) territory. **B** After PCA occlusion (ischemic territorial mismatch): Progressive proximal occlusion of the PCA leads to selective hemodynamic compromise of vulnerable superficial leptomeningeal collaterals supplying the MCA territory. This results in critical hemodynamic compromise confined to the MCA territory, manifested by the emergence of the FLAIR ivy sign and increased susceptibility to ischemia or infarction, while other territories remain relatively preserved. **C** After revascularization (acute phase): Immediately after extracranial-to-intracranial bypass surgery, direct restoration of blood flow to the MCA territory may result in transient focal hyperperfusion in the early postoperative phase. **D** After revascularization (chronic phase): Following revascularization, cerebral hemodynamics improved, accompanied by a reduction in the FLAIR ivy sign, reflecting the hemodynamic impact of surgical intervention

**Fig. 2:**
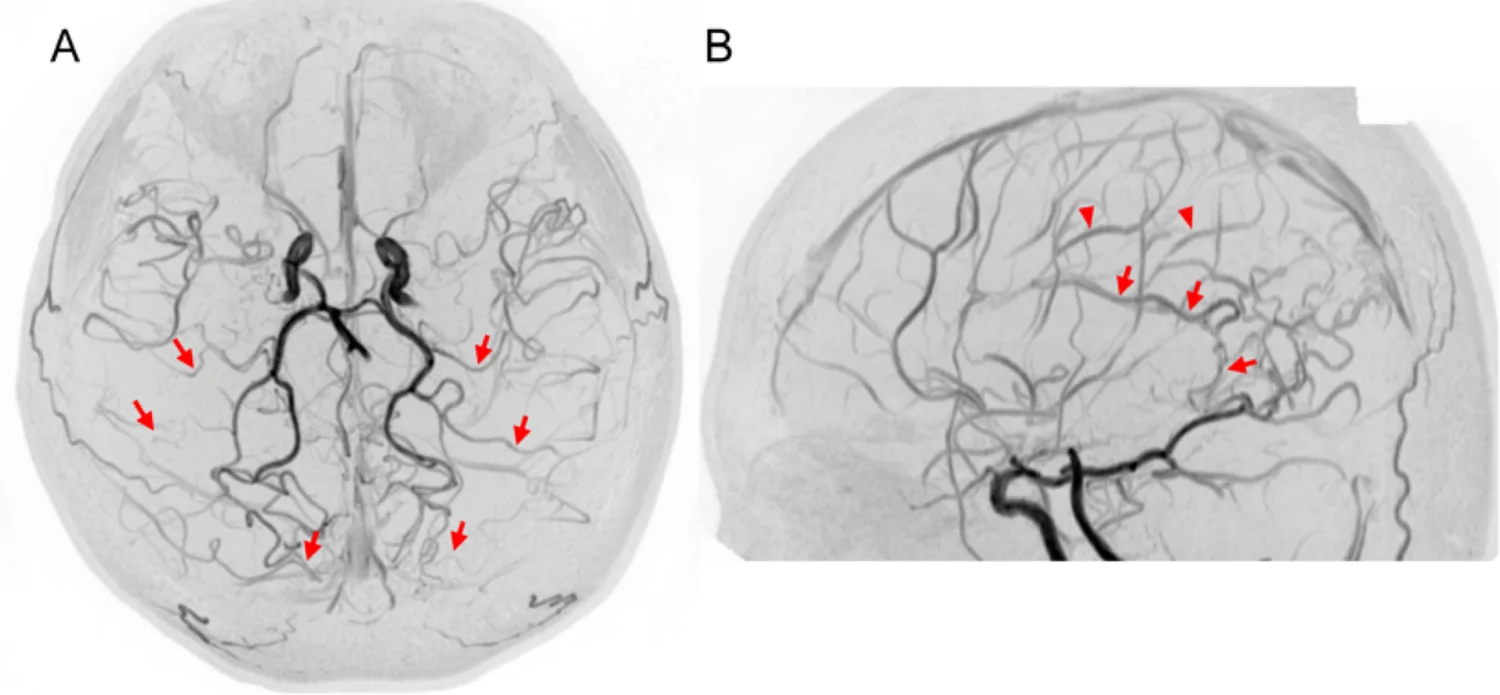
Anatomy of posterior cerebral artery (PCA)–derived superficial and deep leptomeningeal collateral pathways in moyamoya disease. A middle-aged woman presented with bilateral occlusive changes at the terminal portions of the internal carotid arteries, without steno-occlusive changes in posterior cerebral artery (PCA). **A** Axial maximum intensity projection (MIP) MR angiography illustrates superficial leptomeningeal collateral pathways. Distal branches of the PCA extend predominantly toward the middle cerebral artery (MCA) territory (arrows), serving as surrogate markers of this superficial collateral pathway. **B** Sagittal MIP MR angiography demonstrates deep collateral pathways. The posterior pericallosal artery (arrows) is visualized as the primary anatomical conduit of this pathway, extending branches toward the anterior cerebral artery (ACA) territory (arrowheads)

PCA-derived leptomeningeal collateral circulation comprises two functionally distinct pathways [2, 4–6]. Proximal deep collaterals supply the anterior cerebral artery (ACA) territory through a shorter pathway via the posterior pericallosal artery, whereas distal superficial collaterals must traverse substantially longer cortical distances to reach the middle cerebral artery (MCA) territory (Fig. 1A). Steno-occlusive changes preferentially develop at the proximal segment of the PCA and are often accompanied by moyamoya vessel formation, including anastomoses between the medial posterior choroidal artery and the posterior pericallosal artery. As a result, with progressive PCA steno-occlusive changes, superficial collateral pathways may become insufficient earlier than the more resilient deep collaterals. Consequently, ischemia or infarction associated with PCA steno-occlusion preferentially affects the MCA territory (Fig. 1B), while the anatomical PCA and ACA territories remain relatively preserved until PCA lesions become extremely advanced [4, 5]. Representative MR angiographic anatomy of PCA-derived superficial and deep leptomeningeal collateral pathways in moyamoya disease is shown in Fig. 2.

The fluid-attenuated inversion recovery (FLAIR) ivy sign reflects slow flow within maximally dilated cortical vessels that develop as a compensatory response to hemodynamic compromise [7]. In this context, ischemic territorial mismatch refers to a discrepancy between the site of PCA involvement and the distribution of ischemic changes, such as preferential ischemia in the MCA territory despite PCA steno-occlusion.

In this pictorial essay, based on this hemodynamic framework, we visually present a hypothesis-generating conceptual framework of ischemic territorial mismatch due to PCA occlusion and demonstrate the interpretation and clinical utility of the FLAIR ivy sign by integrating schematic illustrations with representative cross-sectional imaging findings and longitudinal angiographic and imaging findings obtained before and after revascularization surgery (Fig. 1C, D).

## Illustrative case and imaging findings

### Case 1: pediatric cross-sectional presentation

**Fig. 3:**
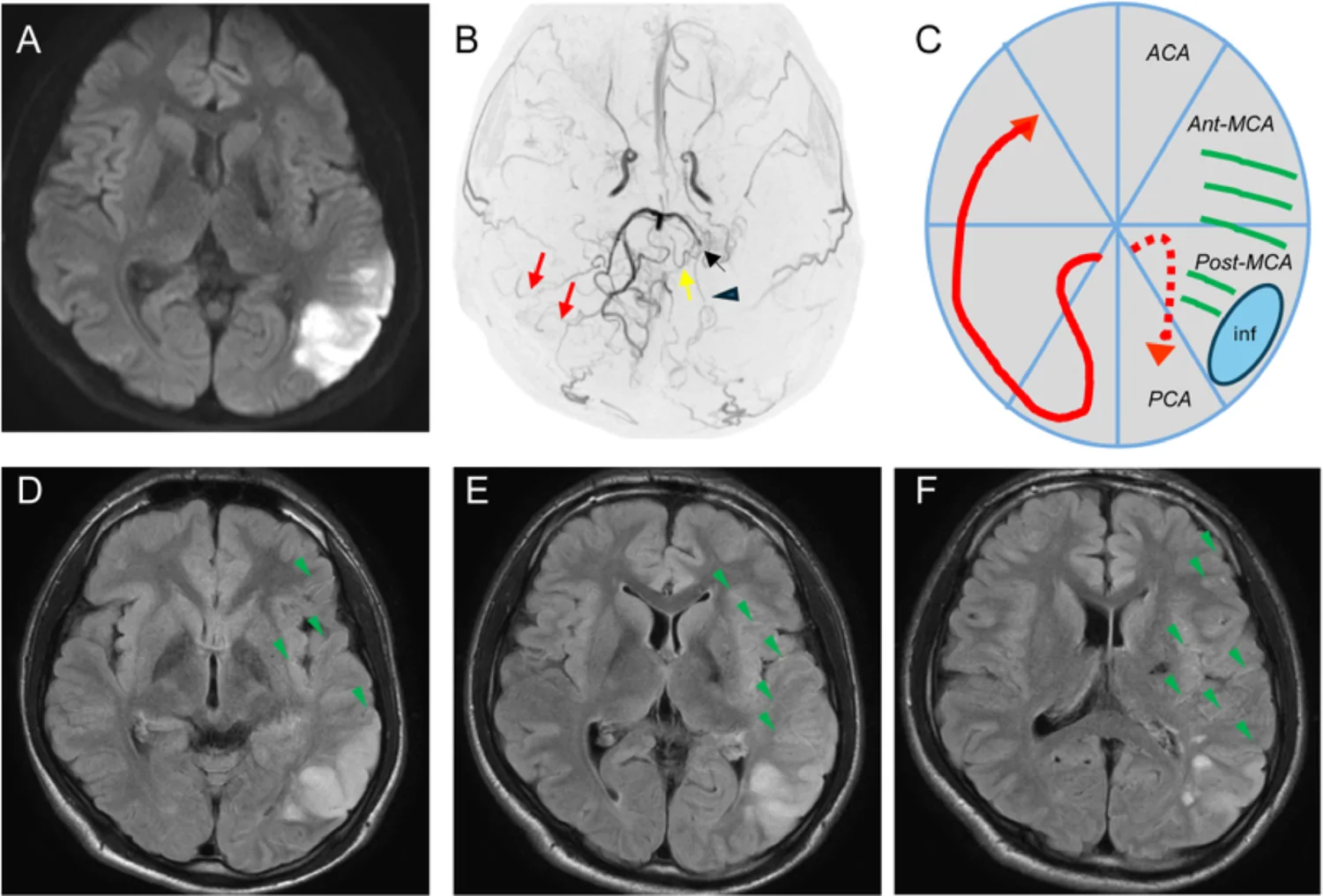
Case 1: Representative presentation of ischemic territorial mismatch in a pediatric patient with moyamoya disease. **A** Diffusion-weighted imaging (DWI) demonstrates acute infarction predominantly involving the posterior portion of the left middle cerebral artery (post-MCA) territory, while the anatomical posterior cerebral artery (PCA) territory is spared. **B** Axial maximum intensity projection (MIP) MR angiography shows severe bilateral internal carotid artery (ICA) steno-occlusive changes. The left PCA is occluded proximally (black arrow), whereas the distal PCA trunk remains visualized (arrowhead), likely reflecting prior compensatory dilatation that had supported collateral flow to the MCA territory. An enlarged medial posterior choroidal artery (yellow arrow) arises from the proximal left PCA, suggesting involvement of deep collateral pathways related to the anterior cerebral artery (ACA) territory. The right PCA remains patent, preserving compensatory superficial collateral supply to the anterior circulation (red arrows). **C** A schematic diagram illustrates the mechanism of ischemic territorial mismatch. Proximal PCA occlusion in the left hemisphere (red dashed arrow) results in functional insufficiency of PCA-derived superficial leptomeningeal collaterals, leading to infarction in the dependent post-MCA territory (blue shaded area; “inf”). In contrast, a patent PCA in the right hemisphere (red solid arrow) preserves superficial collateral supply to the anterior circulation. **D**–**F** Sequential axial fluid-attenuated inversion recovery (FLAIR) images show a prominent ivy sign (green arrowheads) distributed across the left MCA territory, reflecting sluggish cortical flow within dilated pial vessels in the setting of reduced superficial collateral supply, as illustrated in panel C.

A pediatric patient with moyamoya disease presented with ischemic stroke in the left hemisphere. MR angiography revealed bilateral internal carotid artery stenosis and proximal occlusion of the left PCA (Fig. 3B). Paradoxically, diffusion-weighted imaging demonstrated acute infarction predominantly involving the middle cerebral artery (MCA) territory, whereas the anatomical PCA territory was entirely spared (Fig. 3A). This characteristic pattern is schematically illustrated in Fig. 3C, highlighting selective vulnerability of the MCA territory despite PCA involvement. FLAIR imaging revealed a prominent ivy sign localized specifically to the MCA territory (Fig. 3D–F), reflecting critically sluggish flow within maximally dilated cortical vessels that develop to compensate for reduced PCA-derived superficial leptomeningeal collateral flow. In contrast, the contralateral hemisphere showed no PCA steno-occlusive changes, well-developed superficial leptomeningeal collaterals on MR angiography, and no evidence of an ivy sign.

## Case 2: adult longitudinal evolution

**Fig. 4:**
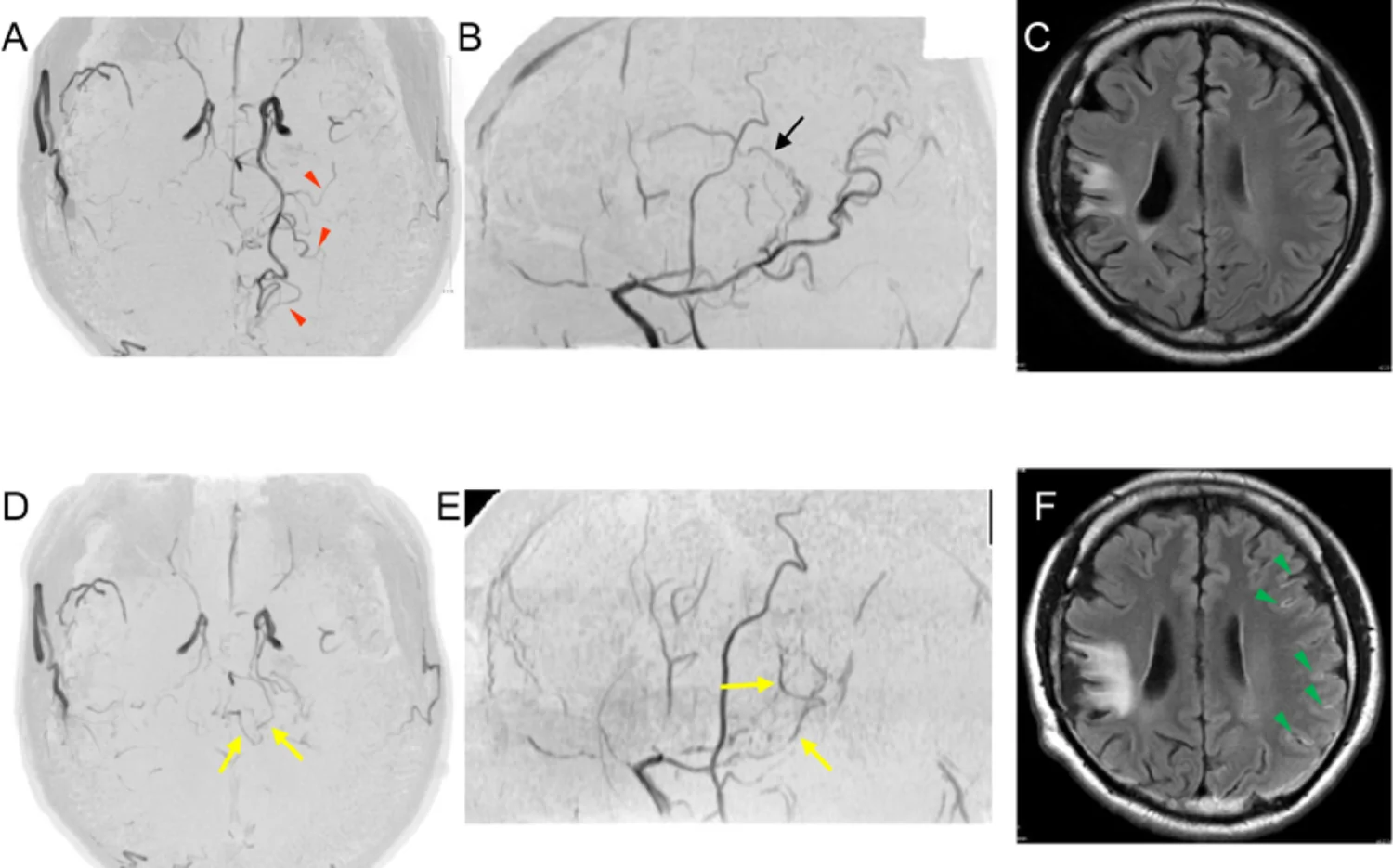
Case 2: Longitudinal imaging evidence of ischemic territorial mismatch associated with progressive posterior cerebral artery occlusion (Phase 1–2). **A**–**C** Phase 1 (stable postoperative phase): Baseline findings obtained during a 10-year asymptomatic follow-up period after right-sided revascularization surgery. The operated right hemisphere shows no ischemic symptoms and no FLAIR ivy sign, consistent with stable postoperative perfusion. **A** Axial maximum intensity projection (MIP) MR angiography demonstrates a patent left posterior cerebral artery (PCA). Robust visualization of distal PCA branches (arrowheads) indicates well-developed PCA-derived superficial leptomeningeal collaterals that effectively compensated for anterior circulation insufficiency. **B** Sagittal MIP MRA illustrates collateral pathways arising from the left PCA. Prominent visualization of the posterior pericallosal artery (black arrow) together with well-visualized distal PCA branches reflects preserved collateral capacity through anatomically distinct pathways. **C** Axial fluid-attenuated inversion recovery (FLAIR) image shows no evidence of the ivy sign, indicating that PCA-derived collateral flow maintained adequate perfusion to the anterior circulation. **D**–**F** Phase 2 (symptomatic phase with PCA occlusion and emergence of the FLAIR ivy sign): Imaging findings obtained at the onset of recurrent transient ischemic attacks localized to the left hemisphere. **D** Axial MIP MR angiography reveals occlusion of the proximal left PCA. In contrast to the stable phase, distal PCA signals are no longer visualized, suggesting collapse of superficial leptomeningeal collaterals that had previously supplied the middle cerebral artery (MCA) territory. This abrupt loss of distal collateral flow marks the hemodynamic transition toward ischemic territorial mismatch. **E** Sagittal MIP MRA demonstrates differential involvement of collateral pathways. While superficial leptomeningeal collaterals are markedly reduced, the medial posterior choroidal artery (yellow arrows) remains dilated and well visualized, suggesting relative preservation of deep collateral pathways to the anterior cerebral artery (ACA) territory. **F** Axial FLAIR image demonstrates new emergence of a prominent ivy sign (green arrowheads) selectively distributed within the left MCA territory, with no corresponding findings in the anterior cerebral artery (ACA) or PCA territory. This selective distribution provides visual evidence of ischemic territorial mismatch and reflects a state of critical hemodynamic compromise due to PCA-derived superficial collateral insufficiency

**Fig. 5:**
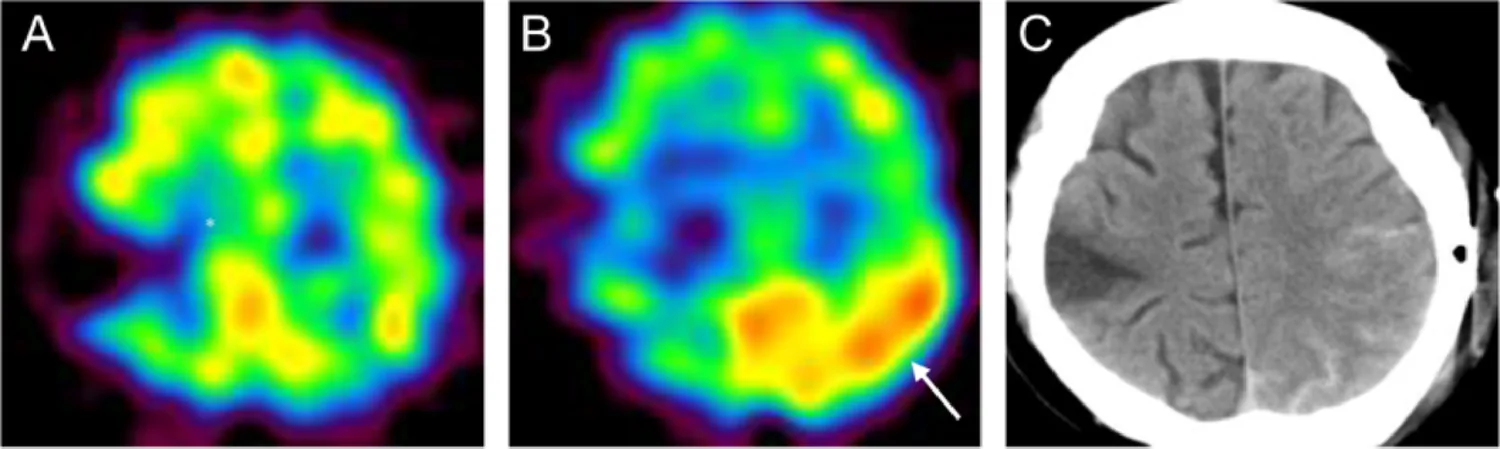
Case 2: Perioperative perfusion changes and postoperative hyperperfusion (Phase 3). **A** Preoperative 123I-IMP SPECT shows reduced cerebral perfusion in the bilateral middle cerebral artery (MCA) territories. A focal area of markedly decreased uptake in the right hemisphere (asterisk) corresponds to a chronic lesion related to prior hyperperfusion injury after right-sided revascularization. **B** 123I-IMP SPECT obtained on postoperative day 2 after left-sided superficial temporal artery–middle cerebral artery (STA–MCA) bypass demonstrates focal hyperperfusion in the posterior portion of the left MCA territory (arrow). **C** Non-contrast computed tomography reveals a localized convexity subarachnoid hemorrhage in the left hemisphere, likely associated with the focal hyperperfusion shown in Panel B.

**Fig. 6:**
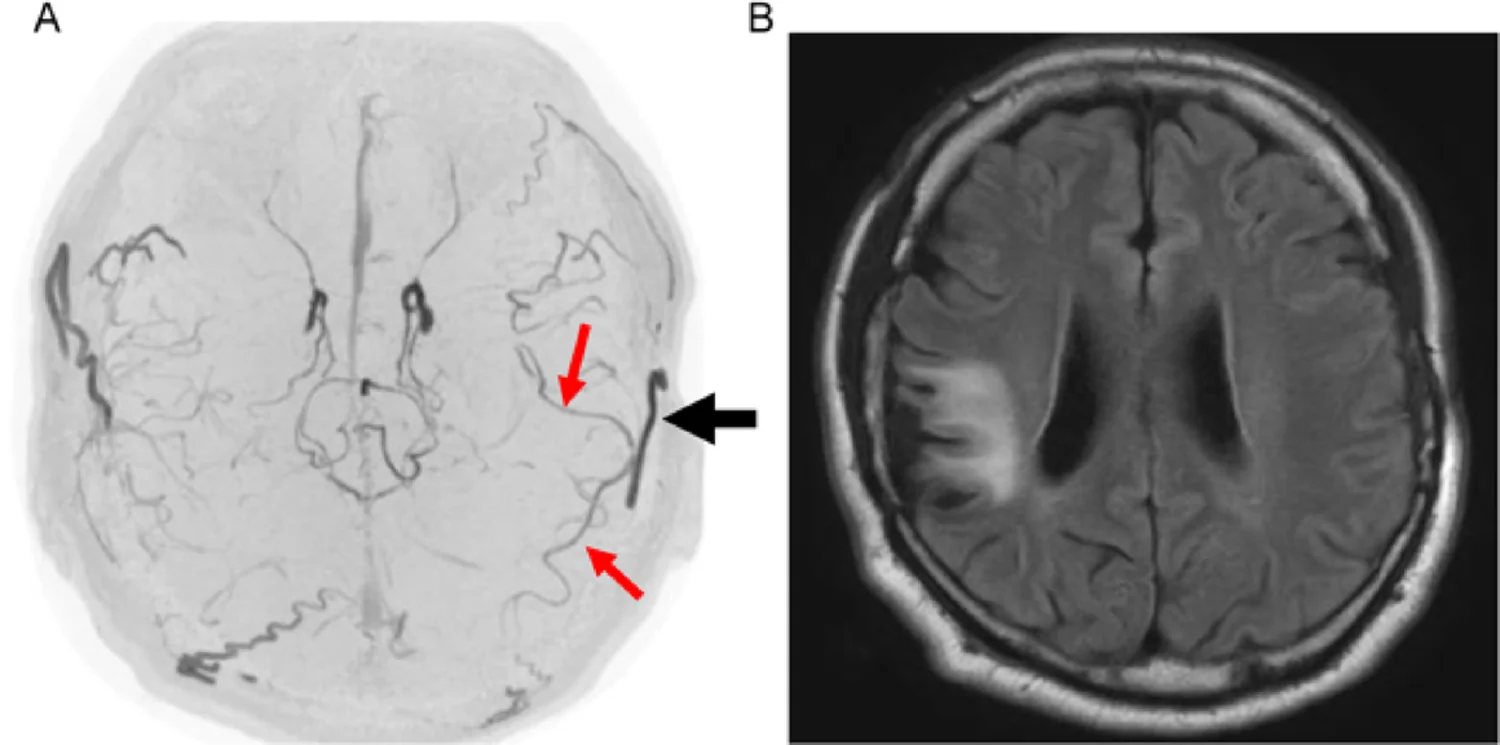
Case 2: Postoperative follow-up imaging demonstrating hemodynamic stabilization after revascularization and resolution of the FLAIR ivy sign (Phase 4). (A) Axial maximum intensity projection (MIP) MR angiography obtained five months after bypass surgery demonstrates good patency of the bypass graft (black arrow) with extracranial-to-intracranial collateral flow supplying the left cerebral hemisphere (red arrows), consistent with functional compensation for previously compromised posterior cerebral artery–derived superficial collateral pathways. (B) Follow-up fluid-attenuated inversion recovery (FLAIR) imaging shows resolution of the previously observed ivy sign in the left hemisphere. In the absence of new ischemic lesions, this finding is consistent with stabilization of cerebral hemodynamics after revascularization.

A man in his 40s with moyamoya disease demonstrated longitudinal hemodynamic evolution following right-sided revascularization bypass surgery. For clarity, the clinical and imaging course is described in four phases: Phase 1 (stable postoperative phase) and Phase 2 (symptomatic phase with PCA occlusion and emergence of the FLAIR ivy sign), corresponding to the findings shown in Fig. 4; Phase 3 (early postoperative phase following revascularization), corresponding to Fig. 5; and Phase 4 (recovery phase), corresponding to Fig. 6. These four phases collectively recapitulate the hemodynamic states illustrated in Fig. 1.

During the initial stable postoperative phase (approximately 9 years after the initial revascularization), the operated right hemisphere remained asymptomatic and free of the FLAIR ivy sign, consistent with restored and stable perfusion. At the same time, MR angiography confirmed patency of the left PCA (Fig. 4A, B), and FLAIR imaging showed no evidence of an ivy sign in the left hemisphere (Fig. 4C), indicating preserved PCA-derived collateral flow.

The patient subsequently entered a symptomatic phase approximately 10 years after the initial surgery, characterized by recurrent transient ischemic attacks (TIAs). MR angiography revealed new proximal occlusion of the left PCA (Fig. 4D, E), accompanied by the simultaneous emergence of a prominent ivy sign across the left MCA territory on FLAIR imaging (Fig. 4F). These findings were consistent with selective insufficiency of PCA-derived superficial leptomeningeal collaterals, while deeper collateral pathways were relatively preserved.

Left-sided superficial temporal artery–middle cerebral artery (STA–MCA) bypass surgery was then performed during this symptomatic phase. Early postoperative imaging obtained on postoperative day 2 demonstrated focal hyperperfusion in the left MCA territory with a localized convexity subarachnoid hemorrhage (Fig. 5A–C) [8].

Subsequent imaging obtained five months after bypass surgery confirmed excellent bypass patency and disappearance of the ivy sign (Fig. 6A, B), indicating that the FLAIR ivy sign is a dynamic and reversible marker of hemodynamic compromise in the left MCA territory following revascularization surgery. In contrast, the contralateral (right) hemisphere, which had undergone prior revascularization, showed no ivy sign despite persistent PCA occlusion, corresponding to a stable postoperative hemodynamic state (Fig. 1D).

In contrast to Case 1, in which revascularization was performed after completed cerebral infarction, this patient underwent revascularization after the emergence of the FLAIR ivy sign but before infarction developed. Taken together, the longitudinal imaging findings in this case recapitulate the four hemodynamic phases illustrated in Fig. 1 and demonstrate the dynamic evolution of PCA-related ischemic territorial mismatch before and after revascularization.

## Integrated interpretation of the FLAIR ivy sign and ischemic territorial mismatch

The findings of this pictorial essay represent an illustrative, hypothesis-generating conceptual framework rather than definitive mechanistic evidence. The cases demonstrate that the FLAIR ivy sign reflects dynamic and reversible hemodynamic compromise related to PCA-derived superficial collateral insufficiency, in the setting of ischemic territorial mismatch.

Before PCA steno-occlusive changes develop, both superficial and deep PCA-derived collateral pathways are recruited to compensate for anterior circulation insufficiency [2]. Because superficial collaterals supplying the MCA territory traverse longer cortical distances, they may become insufficient earlier than the more resilient deep pathways supplying the ACA territory. Consequently, deep collateral pathways may remain relatively preserved until more advanced stages of PCA involvement, contributing to relative sparing of the ACA and PCA territories in the earlier phase.

This conceptual framework is further supported by prior observational data. In a cohort of 66 patients (132 hemispheres) with moyamoya disease, the presence of cerebral infarction was not associated with the degree of ICA involvement but was closely related to PCA steno-occlusive changes [1]. In addition, in a cohort of 69 untreated patients (137 hemispheres), infarctions in the MCA territory were frequently associated with PCA steno-occlusive changes, occurring in 20 of 25 (80%) anterior MCA infarctions and 35 of 37 (95%) posterior MCA infarctions [2]. These findings, together with cerebral perfusion studies demonstrating more pronounced hemodynamic compromise in the MCA territory [4] and the preferential distribution of the FLAIR ivy sign in the MCA territory [7], may support the concept that insufficiency of superficial collateral pathways predominantly affects the MCA territory, a hemodynamically vulnerable region in the setting of PCA-dependent collateral circulation.

Although PCA involvement tends to occur earlier in pediatric patients and later in adults after prolonged dependence on PCA-derived collateral flow [9], the resulting hemodynamic consequence is similar across age groups [4, 10], characterized by deterioration of cerebral perfusion reflected by the FLAIR ivy sign [11, 12].

## Clinical implications of the FLAIR ivy sign for follow-up and surgical management

Recognition that PCA progression precipitates MCA-territory hemodynamic compromise is important for long-term management [13]. In this context, the FLAIR ivy sign serves as a noninvasive marker of impaired cerebrovascular reserve [7], and its preoperative presence has been associated with an increased risk of postoperative hyperperfusion [14, 15].

In this context, the FLAIR ivy sign may provide a useful framework for interpreting two distinct mechanisms underlying progression of PCA steno-occlusive changes: primary progression of the disease process and secondary changes related to reduced hemodynamic demand after successful revascularization or infarction, as previously suggested [16]. This distinction has important clinical implications for follow-up and surgical decision-making. Primary progression may be associated with ongoing hemodynamic compromise requiring further intervention, whereas secondary changes may not necessarily indicate active ischemic risk.

Although this framework is derived from illustrative cases, the hemodynamic states illustrated in Fig. 1 may provide a useful conceptual model for categorizing hemispheric hemodynamic conditions in moyamoya disease. From the perspective of ischemic territorial mismatch associated with PCA involvement, individual hemispheres may be interpreted as approximating one of these states, depending on the degree of collateral dependence and progression of PCA steno-occlusive changes. This interpretation is further supported by the contralateral hemispheres in the presented cases, which demonstrate distinct hemodynamic states within the same patients. This model may facilitate interpretation of the dynamic redistribution of perfusion territories and provide a clinically useful perspective for understanding heterogeneous and evolving patterns of ischemia in moyamoya disease.

In addition, the dynamic behavior of the FLAIR ivy sign, including its emergence and resolution, may provide further insight into these mechanisms and enable assessment of the evolving hemodynamic state over time, offering noninvasive information on superficial collateral insufficiency not captured by vascular morphology alone and supporting longitudinal follow-up.

The FLAIR ivy sign is not a specific marker of superficial collateral insufficiency. As previously reported, the degree of the FLAIR ivy sign is associated with impaired cerebrovascular reserve, and it can be observed even in the absence of PCA steno-occlusive changes when cerebrovascular reserve is reduced [7]. Its appearance may reflect a broader state of hemodynamic disturbance and can be influenced by multiple factors, including metabolic demand and alternative collateral pathways. Accordingly, its interpretation should be made in the appropriate clinical and imaging context. Importantly, its clinical value lies primarily in its dynamic and longitudinal behavior, as illustrated in Case 2, rather than in static diagnostic performance at a single time point.

From a broader perspective, unlike ischemic conditions in other cerebrovascular diseases, ischemia in moyamoya disease is dynamic and evolves in response to changes in collateral circulation. In particular, PCA steno-occlusive changes may compromise collateral pathways that had previously compensated for anterior circulation insufficiency, leading to redistribution of perfusion territories. This mechanism may underlie the spatially discordant patterns of ischemia observed in moyamoya disease. In this context, ischemic territorial mismatch associated with PCA involvement, as illustrated by the FLAIR ivy sign in the present study, may represent a characteristic feature of moyamoya disease.

## Conclusion

This integrated interpretation provides a plausible conceptual explanation for ischemic presentations associated with PCA involvement in moyamoya disease. The FLAIR ivy sign reflects superficial collateral-related hemodynamic compromise and highlights the dynamic and evolving nature of ischemic distribution associated with PCA involvement in moyamoya disease, which may represent a characteristic feature of this disease.

## Data Availability

Data will be made available on reasonable requests.
